# Experiences with the standardized classification of surgical complications (Clavien-Dindo) in general surgery patients

**DOI:** 10.1007/s10353-018-0551-z

**Published:** 2018-07-24

**Authors:** M. Bolliger, J.-A. Kroehnert, F. Molineus, D. Kandioler, M. Schindl, P. Riss

**Affiliations:** Division of General Surgery, Department of Surgery, Medical University of Vienna, Waehringer Guertel 18–20, 1090 Vienna, Austria

**Keywords:** Postoperative complications, General surgery, Morbidity, Retrospective study, University hospital

## Abstract

**Background:**

The standardized Clavien-Dindo classification of surgical complications is applied as a simple and widely used tool to assess and report postoperative complications in general surgery. However, most documentation uses this classification to report surgery-related morbidity and mortality in a single field of surgery or even particular intervention. The aim of the present study was to present experiences with the Clavien-Dindo classification when applied to all patients on the general surgery ward of a tertiary referral care center.

**Methods:**

We analyzed a period of 6 months of care on a ward with a broad range of general and visceral surgery. Discharge reports and patient charts were analyzed retrospectively and reported complications rated according to the most recent Clavien-Dindo classification version. The complexity of operations was assessed with the Austrian Chamber of Physicians accounting system.

**Results:**

The study included 517 patients with 817 admissions, of whom 463 had been operated upon. Complications emerged in 12.5%, of which 19% were rated as Clavien I, 20.7% as Clavien II, 13.8% as Clavien IIIa, 27.6% as Clavien IIIb, 8.6% as Clavien IVa, and 10.3% as Clavien V. No Clavien grade IVb complication occurred within the investigation. Patients having undergone more complex surgery or with higher scores experienced significantly longer lengths of hospital stay.

**Conclusion:**

The Clavien-Dindo classification can easily be used to document complication rates in general surgery, even though this collective was not included in the original validation studies of Clavien et al. and consisted of more heavily impaired patients.

## Main novel aspects

- As far as we know, this is the first study to use the Clavien-Dindo classification in patients from the whole general surgical spectrum.

## Introduction

How can surgeons assess—and for that matter, even improve—their proficiency in an effort to also enhance the outcome and overall experience of and for their patients?

Initially, the probably most important step is to report and examine potential complications and draw lessons from them. Several authors are still in a debate concerning a universal definition of “complications,” including Veen and colleagues’ equation (“a complication is every unwanted development in the illness of the patient or in the treatment of the patient’s illness that occurs in the clinic”) [1], Clavien’s original definition (“complications are unexpected events not intrinsic to the procedure”) [2], and Sokol and Wilson’s variation (“A surgical complication is any undesirable, unintended, and direct result of an operation affecting the patient, which would not have occurred had the operation gone as well as could reasonably be hoped”) [3].

The Clavien-Dindo classification originated in 1992, when it was first introduced under the name of the “T92 score,” as validated on 650 cholecystectomies [2]. This new scoring system offered the advantages of being able to compare results over different time periods within the same institution, compare different institutions, compare surgical and conservative treatments, and document operations and associated complications in a standardized way, and therefore facilitate meta-analyses. In addition, prognostic scores could thus be implemented.

An updated version was presented in 2004, when new subcategories and a separate suffix for permanent disability were introduced. Furthermore, Clavien and Dindo discarded length of stay (LOS) in this new version, since it differed too strongly between hospitals due to internal rules governing when to discharge a patient. This was also shown by Peterson and colleagues in 2002, who compared the lengths of stay following close to half a million coronary artery bypass graft procedures in 587 US hospitals. In this study, even after adjusting for patient risk factors, an inexplicable variety among the postoperative lengths of stays remained [4]. Salmon showed similar results for orthopedic patients in 2013, claiming that a hospital with rapid discharge did not produce inferior outcomes or worse patient feedback [5].

The update also included a survey among 144 surgeons, which showed that it was indeed easy to classify the complication grades, regardless of the physicians’ age or experience. Over 90% of the survey participants voted in favor of introducing this score into clinical routine [6]. In 2009, the same authors published a five-year experience report, in which they asked seven hospitals which were already using the score to rate 11 difficult cases. This query revealed an adequate implementation of their score and an 89% concordance between the centers. The authors concluded that it would be preferable to omit subjective evaluations, such as minor and major complications [7].

Meanwhile, the Clavien-Dindo classification has been used in hospitals around the world and evaluated for various surgeries.

This present study aimed to analyze the implementation of the Clavien-Dindo classification for complications in the general surgery patient ward of a tertiary referral care center (university hospital), offering the entire spectrum of this specialization, which has not yet been documented in the literature.

## Materials and methods

Over the 6‑month period covering April 2010 to September 2010, all patients admitted to one of our patient wards at the Division of General Surgery, Department of Surgery, Medical University of Vienna were included in this study.

The Division of General Surgery in our university hospital consists of the following teams and specializations: colorectal surgery, hepatobiliary surgery, endocrine surgery, upper gastrointestinal (GI) surgery (esophageal and stomach surgery), bariatric surgery, breast surgery, and pancreatic surgery.

The patient data were extracted by reviewing all discharge letters from that period taken from the digital archives.

Overall, 517 patients were admitted over this period, some repeatedly, leading to a total of 817 admissions. These 517 patients underwent 463 operations. The complications of these operations were then rated according to the Clavien-Dindo classification (Table 1). For easier use, the suffix “d” for permanent disability was not drawn upon.

**Table 1:** Clavien-Dindo classification

Grade	Definition
*Grade I*	Any deviation from the normal postoperative course without the need for pharmacological treatment, or surgical, endoscopic, and radiological interventions. Allowed therapeutic regimens are: drugs as antiemetics, antipyretics, analgetics, diuretics and electrolytes, and physiotherapy. This grade also includes wound infections opened at the bedside
*Grade II*	Requiring pharmacological treatment with drugs other than such allowed for grade I complications. Blood transfusions and total parenteral nutrition are also included
*Grade III*	Requiring surgical, endoscopic, or radiological intervention
Grade IIIa	Intervention not under general anesthesia
Grade IIIb	Intervention under general anesthesia
*Grade IV*	Life-threatening complication (including central nervous system complications) requiring IC/ICU management
Grade IVa	Single organ dysfunction (including dialysis)
Grade IVb	Multiorgan dysfunction
*Grade V*	Death of a patient

According to Dindo et al. [6]

*IC* intermediate care, *ICU* intensive care unit

The operations were sorted according to the complexity ranking (eight groups) in the accounting system of the Austrian Chamber of Physicians (Table 2; [8]).

**Table 2:** Operation groups (complexity according to the Austrian Chamber of Physicians)

Operation group	Examples
I	Abscess incisions, secondary sutures, proctoscopy, skin biopsy
II	Excisions of atheromas, fibromas, lipomas, incisions of anal abscesses
III	Toe amputation, small lymph node extirpation, thoracic drainage, colonoscopy
IV	Tracheotomy, appendectomy, hernia operation, colostomy, gastrostomy, ERCP
V	Gastroenterostomy, interventions for recurrent hernia, Cimino fistula, radical varicose vein stripping
VI	Strumectomy, cholecystectomy, splenectomy, hemicolectomy, reduction mammoplasty
VII	Partial pancreatectomy, subtotal colectomy, subsegmental and large liver resections
VIII	Esophageal resection, open surgery of aortic aneurysms, organ transplantation

### Statistical analysis

The statistical analysis was done with SPSS (IBM SPSS Statistics Version 21, International Business Machines Corporation, Armonk, USA). Two-sided Spearman’s rank correlation coefficients were calculated. For patients undergoing more than one surgical procedure, the most complex operation was used for complications. For the correlation analysis, only the first procedure was assessed to guarantee the sample’s independence. A *p*-value < 0.05 was defined as statistically significant.

## Results

This study included 517 patients with 817 admissions. Some patients had more than one admission. Age at admission ranged from 18 to 89, the average age was 58.9 years. The gender ratio was 53.7% male to 46.3% female. Length of stay (LOS) ranged from 0 to 74 days (median 2 days, mean 4.66 days), the median was 2 days for male (mean 4.72 days) and 3 days for female patients (mean 4.59 days).

Surgery was performed in 463 patients (56.7%), 257 patients (31.4%) received chemotherapy, and 97 patients (11.9%) were admitted for conservative or diagnostic measures.

### Length of stay

In the group of operated patients, the mean LOS was 7.02 days (0 to 74 days, median 4 days; 2.36 days longer than the entire cohort; patients with zero days LOS were day-admissions).

The most common procedures were abdominal and gastrointestinal surgery (264/463, 57%), followed by general operations (68/463, 14.7%) and breast surgery (36/463, 7.8%).

The patients who stayed the longest on average underwent surgery of the urinary tract, i.e., mostly kidney transplantations (mean LOS 14.17 days). Those were followed by patients given endoscopic interventions (mean LOS 9.81 days), abdominal/GI operations (mean LOS 8.10 days), lung/thoracic surgery (mean LOS 7 days), urological procedures (mean LOS 6 days), and vascular surgery (mean LOS 5.8 days).

### Complexity of surgery

Concerning the complexity of the procedures according to the accounting system of the Chamber of Physicians, the most common operations were of complexity grade 4 (151/463, 32.6%), followed by grade 5 (69/463, 14.9%), grade 6 (55/463, 11.9%), and grade 3 (45/463, 9.7%).

Comparing with complexity, the highest LOS scores were identified in the patients of group VIII operations (mean 13.93 days), followed by group VII (mean 10.74 days), and group VI (mean 9.29 days). LOS by complexity is shown in Table 3.

**Table 3:** Complexity (according to operation group) and length of stay

Complexity grade	*n* (%)	Mean LOS (days)
I	41 (8.9)	3.59
II	31 (6.7)	4.00
III	45 (9.7)	5.22
IV	151 (32.6)	5.74
V	69 (14.9)	7.45
VI	55 (11.9)	9.29
VII	43 (9.3)	10.74
VIII	28 (6)	13.93
Total	462 (100)	7.02

*LOS* length of stay

### Mortality

The rate of mortality (= Clavien V) in the operated cohort was 6/58 patients (10.3%; 1.3% of all operated patients). Of those subjects, one had a cholangiocellular carcinoma which was operated by performing hemihepatectomy, liver segment resection, and lymph node dissection. The patient died of sepsis after postoperative liver failure in an ICU. The remaining five patients had recurrent pancreatic carcinoma and received only palliative surgery, such as gastroenterostomy, biliodigestive anastomosis, endoscopic retrograde cholangiopancreatography, embolization, or explorative laparotomy. Those five patients all died of multiorgan failure due to primary disease progression.

### Complications

According to the Clavien-Dindo classification, 11/58 (19%; 2.4% of all patients) of the complications were rated as Clavien I, 12 (20.7%; 2.6% of all) as Clavien II, 8 (13.8%; 1.7% of all) as Clavien IIIa, 16 (27.6%; 3.5% of all) as Clavien IIIb, 5 (8.6%; 1.1% of all) as Clavien IVa, and 6 (10.3%; 1.3% of all) as Clavien V. No Clavien grade IVb complication occurred over the period under investigation. The most common types of surgery with complications are shown in Table 4.

**Table 4:** Most frequent operations and complications

Operation	*n*	Complications
External hernia repair	54	3 (2 × I, IIIb)
Cholecystectomy	32	2 (II, IIIb)
Breast-conserving surgery	27	3 (II, IIIa, IIIb)
Biopsy	24	0
Appendectomy	21	1 (IIIb)
Portacath implantation	21	0

**Table 5:** Frequency of Clavien-Dindo grades and length of stay

Grade	Total	Percentage overall (%; *n* = 462)	Of complications (*n* = 58; %)	LOS (days)
None	405	87.5	–	5.42
I	11	2.4	19.0	11.00
II	12	2.6	20.7	13.92
IIIa	8	1.7	13.8	16.63
IIIb	16	3.5	27.6	19.30
IVa	5	1.1	8.3	45.80
IVb	–	–	–	–
V	6	1.3	10.3	16.67

*LOS* length of stay

Correlating LOS and complication, Clavien IVa patients had a mean LOS of 45.8 days, those with Clavien IIIb 19.3 days, those with Clavien V 16.67 days, and those with Clavien IIIa 16.63 days. Patients without complications were discharged after an average of 5.42 days (Table 5).

Neither the Spearman analysis nor the Pearson analysis showed a correlation between organ groups and LOS. The Spearman analysis, however, identified a correlation between complexity grade and LOS (rank correlation co-efficient 0.509, *p* = 0.01).

Correlating the Clavien-Dindo grades with LOS, the Pearson coefficient was 0.512 (*p* = 0.01).

These results show that both more complex operations and higher complication grades result in longer LOS.

## Discussion

The Clavien-Dindo classification is easy to use and has been clinically validated. Furthermore, it has increasingly gained in popularity since it was first published. PubMed searches for “Clavien-Dindo” ranked by year show a distinct increase over the past years, specifically the past decade (1 citation in 2009, 6 in 2010, 28 in 2011, 5 in 2012, 87 in 2013, 164 in 2014, 251 in 2015, 36 in 2016, 524 in 2017).

Other specializations apart from general surgery have consecutively implemented this classification for their own surgical catalogues. For example, Naumann and collaborators brought traumatologists from three centers together to adapt the classification to their needs under the name of Adapted Clavien-Dindo in Trauma (ACDiT), thus being able to classify both surgically and conservatively treated patients [9]. The Division of Plastic Surgery, Medical University of Graz, used the classification to assess their elective reduction mammoplasties in a large retrospective cohort [10]. The European Association of Urology validated the use of the Clavien-Dindo classification by having urologists rate complications in given cases, 57% of whom considered the classification to be adequate in grading postoperative complications. The authors, however, pointed out that another classification would be needed for intraoperative complications [11].

The present analyses demonstrated that the Clavien-Dindo classification can be simply applied, even in an assorted cohort of patients. Patients with higher Clavien scores (more severe complications) had significantly longer LOS, as did patients with more complex surgeries, quite similar to the publication from Clavien and Dindo in 2004 [6]. However, no correlation was identified between LOS and “organ groups.”

As mentioned above, the classification only describes postoperative complications in its five grades. It disregards pre-existing conditions and comorbidities which, apart from surgical proficiency, play a substantial role in any complication. Veltkamp et al. implemented a predictive score using 11 variables for serious complications [12]. The variables included age, pulmonary disease, urgency status, and American Society of Anesthesiologists (ASA) status. Based on these variables, the authors predicted a twofold risk for serious complications in patients undergoing major surgery. The definition of “major surgery” was similar to the levels VI to VIII in this study, indicating the need to portray comorbidities in complication scales. Accordingly, four of five patients who passed away in the present study died due to progression of their primary disease and not as a result of surgery or complications.

Kazaryan et al. proposed to combine intra- and postoperative complications in a “perioperative adverse event” category. Including events leading to no postoperative morbidity, grade I would nevertheless be noted in order to not be repeated (“near-miss” situations). Grade II would bear further consequences for the patients, e.g., the need for conversion during a primary laparoscopic operation. Finally, grade III would bear significant consequences, yet with delayed reintervention [13].

Describing perioperative instead of merely postoperative complications could also help to assess a given surgeon’s quality. However, according to the authors, especially grade I complications are rarely reported. In this study, 11 patients (2.3%) had grade I complications according to the Clavien-Dindo classification.

A variety of classifications have emerged over the past years. For example, the Accordion scale was developed by Strasberg and colleagues, who argued that authors tend to use numeric grading for complications rather than descriptions of the complications themselves and presented a tabular reporting system [14].

Another rather complicated model is the US National Cancer Institute Common Terminology Criteria (NCI CTC) classification. In its most recent modification, the Patient-Reported Outcomes Common Terminology Criteria for Adverse Events (PRO-CTCAE) categorize complications in various fields, including chemotherapy, poisoning, and psychiatric disorders. This version can be downloaded from the NCI Division of Cancer Treatment and Diagnosis [15]. Patient-reported outcomes are included to compare with clinicians’ assessments of adverse events, which in turn additionally improve the original terminology. This inclusion was seen to be necessary, as there are still substantial differences between the ways in which patients see their own complications and the ways in which physicians would rate them [16]. The PRO-CTCAE have also been validated in different languages [17].

Another widely used tool is the National Veterans Affairs Surgical Quality Improvement Program (NSQIP), originally introduced to predict complications in order to improve surgical quality and reduce costs for the Veterans Affairs Department by comparing expected and actual adverse events [18]. Based on this program, Bilimoria and collaborators established the NSQIP Risk Calculator in 2013 [19]. This calculator proved to accurately predict outcomes, e.g., for Whipple procedures [20] and colorectal surgery [21] amongst others.

Even Dindo and Clavien produced a new scoring system, the Comprehensive Complication Index (CCI), first published in 2013 [22]. As a novelty, this index included the patients’ perception of the severity of their complications as well as the physicians’, and sums up all of the occurring complications, not only the gravest. It also seems to be more responsive to therapy effects and allows a longitudinal assessment of the morbidity. In 2017, they published a prospective study after applying the CCI to their own patients (general surgery) for 1 year [23]. They could show that patients with a higher Clavien-Dindo score also had more complications overall. Comparing the two systems, 24% of the patients now fell into a higher quartile than before and only 2% in a lower quartile. Also, morbidity increased from the date of discharge up to 3 months postoperatively by 13%, so collection of data and outpatient management is necessary in this period of time. The authors suggest the use of both of their scores.

Altogether, these scores seem to be much more complex in their application and may thus be less practicable than the Clavien-Dindo classification.

## Limitations of the study

The patients were not selected, and as we collected the patient data from a university hospital, the subjects also tended to present with more comorbidities than would be the case in a smaller center with more elective surgery. We attempted to depict the complexity of the operations by using the accounting system of the Austrian Chamber of Physicians, which may not be the most precise measurement, although it gave us a reasonable approach to differentiate.

All patient data were retrospectively extracted from discharge letters and electronic records. Even though this was carried out as accurately as possible, we cannot assess and guarantee the quality of primary documentation.

## Conclusion

This is the first study to evaluate the Clavien-Dindo classification in the diverse patient population of a general surgery ward offering a broad spectrum of operations, including transplantation and vascular surgery.

Even though several suitable and clinically validated classifications and predictive scores are available to choose from, we can recommend the Clavien-Dindo classification as an easily applicable and comparable instrument and as a standard in the quality management of a department of general surgery.
